# Porcine circovirus type 2 and porcine reproductive and respiratory syndrome virus alone or associated are frequent intralesional detected viruses in porcine respiratory disease complex cases in Northern Italy

**DOI:** 10.3389/fvets.2023.1234779

**Published:** 2023-08-31

**Authors:** Giulia D’Annunzio, Fabio Ostanello, Luisa Vera Muscatello, Massimo Orioles, Niccolò Jacumin, Nicola Tommasini, Giorgio Leotti, Andrea Luppi, Luciana Mandrioli, Giuseppe Sarli

**Affiliations:** ^1^Isituto Zooprofilattico Sperimentale della Lombardia e dell’Emilia – Romagna “Bruno Ubertini”, Brescia, Italy; ^2^Dipartimento di Scienze Mediche Veterinarie, Università di Bologna, Bologna, Italy; ^3^Dipartimento di Scienze agroalimentari, ambientali e animali, Università di Udine, Udine, Italy; ^4^Boehringer Ingelheim Animal Health Italia SpA, Milano, Italy

**Keywords:** histopathology, PCV2, PRRSV, porcine respiratory disease complex, PRDC, porcine health, Italy

## Abstract

**Methods:**

This study aimed to examine the pathological impact of Porcine Circovirus type 2 (PCV2) and Porcine Reproductive and Respiratory Syndrome Virus (PRRSV) through histological and immunohistochemical analysis of 79 cases of Porcine Respiratory Disease Complex (PRDC) collected from 22 farms in Northern Italy. Lung tissue and several lymphoid organ samples were deployed to associate PCV2-positive stain with Circovirus-associated Diseases (PCVD).

**Results:**

The most common lung lesion observed was interstitial pneumonia, alone or combined with bronchopneumonia. By immunohistochemistry, 44 lungs (55.7%) tested positive for PCV2, 34 (43.0%) for PRRSV, 16 (20.3%) for both viruses and in 17 cases (21.5%) neither virus was detected. Twenty-eight out of 44 (63.6%) PCV2-positive cases had lymphoid depletion or granulomatous inflammation in at least one of the lymphoid tissues examined; thus, they were classified as PCV2 Systemic Diseases (PCV2-SD). In the remaining 16 out of 44 cases (36.4%), PCV2-positive lung lesions were associated with hyperplastic or normal lymphoid tissues, which showed PCV2-positive centrofollicular dendritic cells in at least one of the lymphoid tissues examined. Therefore, these cases were classified as PRDC/PCV2-positive. In the PCV2-positive animals, 42.9% of the PCV2-SD cases (12/28) showed immunohistochemistry (IHC) positivity for PRRSV in the lung tissue, while 25.0% of PRDC/PCV2-positive cases (4/16) showed double positivity for PCV2 and PRRSV.

**Discussion:**

In light of the caseload presented in this study, characterized by the high proportion of PCV2-SD cases alongside the overall respiratory symptomatology, it is imperative to emphasize the crucial role of a comprehensive sampling protocol. This is critical to avoid underestimating the harm caused by PCV2 in farms, particularly with respect to the systemic form of the disease. PCV2 and PRRSV remain the primary infections associated with PRDC in Italy that can significantly impact farm health and co-infections in the field can worsen the pathology, thus the selection of appropriate preventive measures is critical.

## 1. Introduction

The term “Porcine Respiratory Disease Complex” (PRDC) refers to a multifactorial disease that arises from a combination of viral, bacterial and, less frequently, parasites, environmental, managerial, and genetic factors, leading to the development of pneumonia in pigs (1). The polymicrobic nature of PRDC presents a formidable diagnostic challenge as different infectious agents affecting the respiratory system often exhibit similar clinical signs (2). This can make it problematic to determine which of the many pathogens involved in PRDC are primarily responsible for respiratory disease. The complexity of interactions between pathogens and their ubiquity further complicate the study, prevention, and control of PRDC, as well as the identification of their direct involvement in the disease development and their correlation with lesions (1, 3, 4).

In suspected PRDC, a range of direct and indirect diagnostic tests can detect pathogens during an infection or monitor infections in a herd, respectively (5). Direct diagnosis of viral diseases is performed using molecular techniques such as Polymerase Chain Reaction (PCR), quantitative PCR (qPCR), or Multiplex PCR (3, 6–10). While these biomolecular methods are highly sensitive, they cannot determine the causal relationship between the pathogen and the related lesion; thus, histopathological investigations must be used to objectively determine the role of identified pathogens in the disease (11). Methods such as immunohistochemistry (IHC) and *in situ* hybridization (ISH) allow the co-localization of pathogens and associated lesions, thereby establishing their contribution to the disease (12).

One of the primary pathogens involved in PRDC is Porcine Circovirus type 2 (PCV2), which was first identified as the causative agent of Post-Weaning Multisystemic Wasting Syndrome (PMWS) in the 1990s also in Italy (13). PCV2 has since been linked to respiratory diseases, and histology and immunohistochemistry confirmed their association with histiocytic/granulomatous interstitial lung lesions and with proliferative and necrotizing pneumonia (PNP) often in co-infection with other viral agents (14–16). The respiratory form of PCV2 has been classified as PCV2-associated respiratory disease (17) or PCV2-lung disease (PCV2-LD) (18). However, it is essential to note that the systemic form of PCV2, also known as PCV2-SD, may induce respiratory signs, indicating some overlap between the two forms of PCV2 diseases (18). Despite its recognition as a significant pathogen involved in PRDC (19–22), PCV2-LD is frequently considered a negligible entity in a herd (23, 24). In their study, Ticó et al. (24) established a final diagnosis of PCV2-SD in many cases clinically identified as PCV2-associated PRDC, thanks to the detection of lymphoid depletion associated with PCV2-positive stain by immunohistochemistry in the lymphoid tissues.

It is widely acknowledged that co-infections caused by viral or bacterial pathogens significantly impact the development of Porcine Circovirus-associated Diseases (PCVD). Opriessnig and Halbur (25) and Ouyang et al. (26) have demonstrated that co-infections can increase the efficiency of disease reproduction and can be commonly observed in field settings linked to clinical signs and severe lesions.

In particular, co-infection of Porcine Circovirus type 2 (PCV2) and Porcine Reproductive and Respiratory Syndrome Virus (PRRSV) has been widely reported in respiratory clinical manifestations (27–31).

The combined impact of PCV2 and PRRSV infections leads to an immunosuppressive effect altering the host’s defence mechanism and increasing its susceptibility to secondary bacterial and viral infections (32–35). Considering the exacerbation of clinical signs resulting from the interaction of these two infections, it is imperative to promptly identify and implement effective management strategies for the two viruses.

In light of this, the present study aims to explore the impact of PCV2 and PRRSV in Porcine Respiratory Disease Complex (PRDC) cases in pigs from various farms in Northern Italy, area of the Country with the highest density of pig farms. The examination was carried out on lungs and lymphoid tissues to determine the extent to which the observed respiratory forms could be attributed to respiratory disease associated with PCV2 localized only in the lung or to PCV2 systemic disease. Furthermore, the study investigates the role played by PCV2 and PRRSV in co-infections in different forms of PRDC.

## 2. Materials and methods

The study was conducted on 79 pigs of 5–8 weeks of age from wean-to-finishing and finishing sites of 22 different multi-site productions in Northern Italy. All the pigs died after suffering respiratory clinical signs and underwent necropsy for diagnostic purposes. Seven of these farms had a history of Porcine Reproductive and Respiratory Syndrome Virus (PRRSV) issues, and all farms had prophylactic vaccination strategies for Porcine Circovirus type 2 (PCV2). All pigs underwent diagnostic necropsy procedures, including tissue sampling (lung, mediastinal, tracheobronchial, and superficial inguinal lymph nodes, tonsils, and ileum) and formalin fixation for histopathological analysis.

### 2.1. Histology and immunohistochemistry

Sections (3 μm) of the lung, tonsil, tracheobronchial and mediastinal lymph node, ileum, and superficial inguinal lymph node were stained with hematoxylin–eosin (H&E; Hematoxylin cat# 01HEMH2500; Eosin cat# 01EOY101000; Histo-Line Laboratories, Milan, Italy). Lung lesions were categorized into bronchopneumonia, interstitial pneumonia, proliferative and necrotizing pneumonia (PNP), and fibrinous pleuropneumonia according to the criteria established by Caswell and Williams (36). Lesions in lymphoid tissues were categorized as lymphoid depletion (37) or lymphoid hyperplasia (38). Immunohistochemistry (IHC) on three-micron-thick sections of the same tissues was performed to detect PCV2 antigen and only on lung sections to detect PRRSV antigen. Briefly, the sections were deparaffinized and rehydrated. The endogenous peroxidase was blocked in 3% peroxide for 30 min. Antigen retrieval was performed using protease type XIV at 0.5% in Tris-buffered saline (TBS) for 20 min at 37°C. After washing, blocking of unspecific bindings was carried out for 30 min at room temperature with bovine serum albumin–PBS Tween 20 solution (3%). Primary monoclonal antibodies for PCV2 (Ingenasa, 36A9) and PRRSV (Ingenasa, 1 AC7) were applied on the corresponding slide tissue and were incubated overnight at 4°C. At the end of the primary antibody incubation, the sections were rinsed three times in PBS, followed by a 30 min incubation in Scytek CRF Anti-Polyvalent HRP Polymer and by application to the sections of the final peroxidase substrate, prepared according to manufacturers’ instructions [CRF Anti-Polyvalent HRP Polymer (DAB) Stain Kit, ScyTek Laboratories, CPH080]. Slides were finally counterstained with Papanicolaou’s hematoxylin (Histo-Line Laboratories), and coverslips were applied using Bio Mount mounting medium (Bio-Optica, 05-BMHM100). The specificity of the immunohistochemical stain has been validated by adding, as the primary reagent, an aspecific antibody of the same isotype as the primary antibodies (IgG1). As positive control, samples positive by means of PCR to PCV2 or PRRSV were included.

### 2.2. Diagnostic criteria

The PCV2-positive cases of PRDC involved in this study were categorized based on the current diagnostic criteria for PCVD (23). The cases were considered indicative of PCV2 systemic disease (PCV2-SD) if the lungs were positive for PCV2 antigen and showed associated lymphocyte depletion in lymphoid tissues along with moderate to high amount of the virus. Cases with PCV2-positive lungs but without lymphoid tissue histopathological abnormalities were considered having PRDCs with a PCV2 role, and categorized as PRDC/PCV2-positive, considering that PCV2-LD should no longer be included among PCVDs (23).

## 3. Results

**Figure 1:**
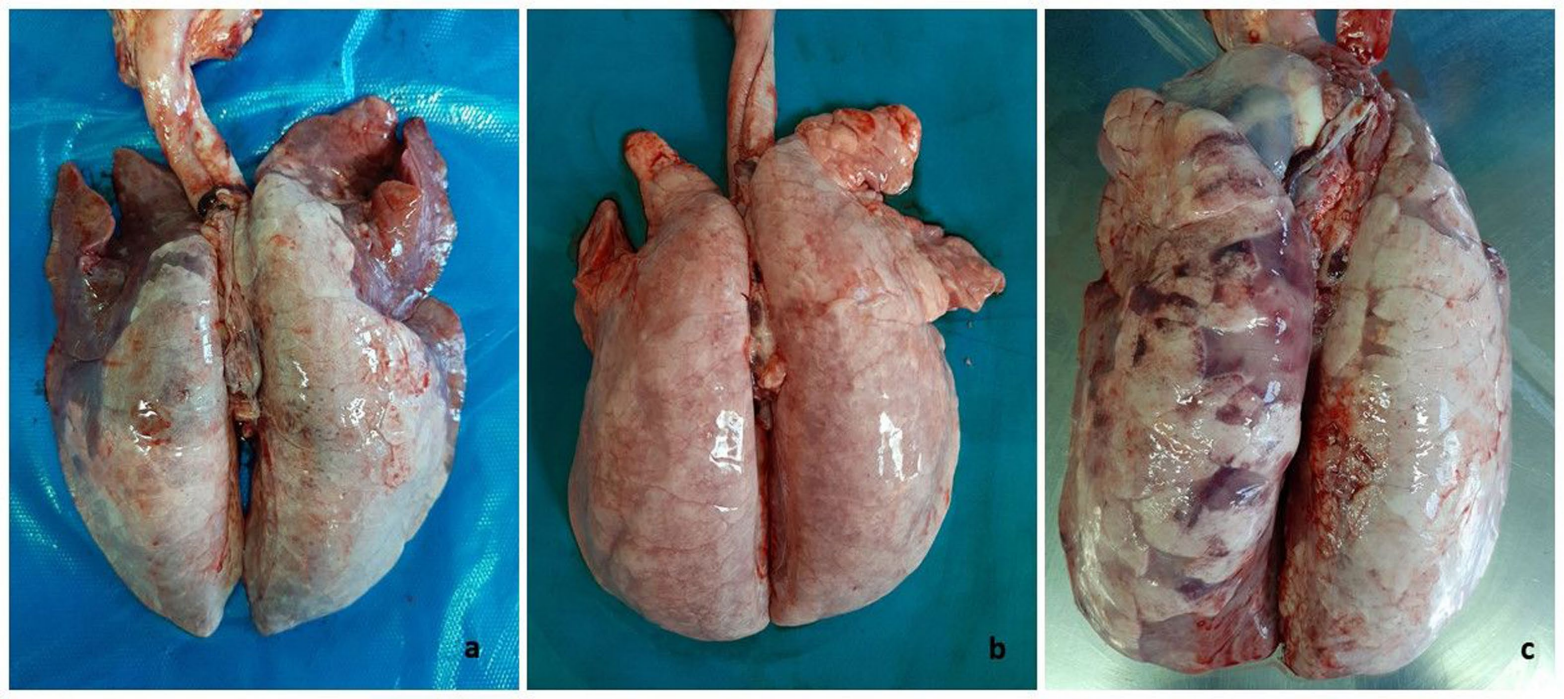
Swine. Lungs. Macroscopic pattern of pneumonia recognized in the caseload. **(A)** Cranioventral dark-red areas of consolidation (bronchopneumonia) associated to uncollapsed basal lobes showing discoloration and edema (interstitial pneumonia), this latter as main lesion appearing in **(B)**. **(C)** Lung with dark areas of consolidation intermingled with interstitial pneumonia in basal lobes.

Seventy-nine cases of pigs with respiratory signs from 22 farms located in Northern Italy were examined in the study. The most common lung lesion observed was interstitial pneumonia, alone or combined with bronchopneumonia (Figure 1).

**Figure 2:**
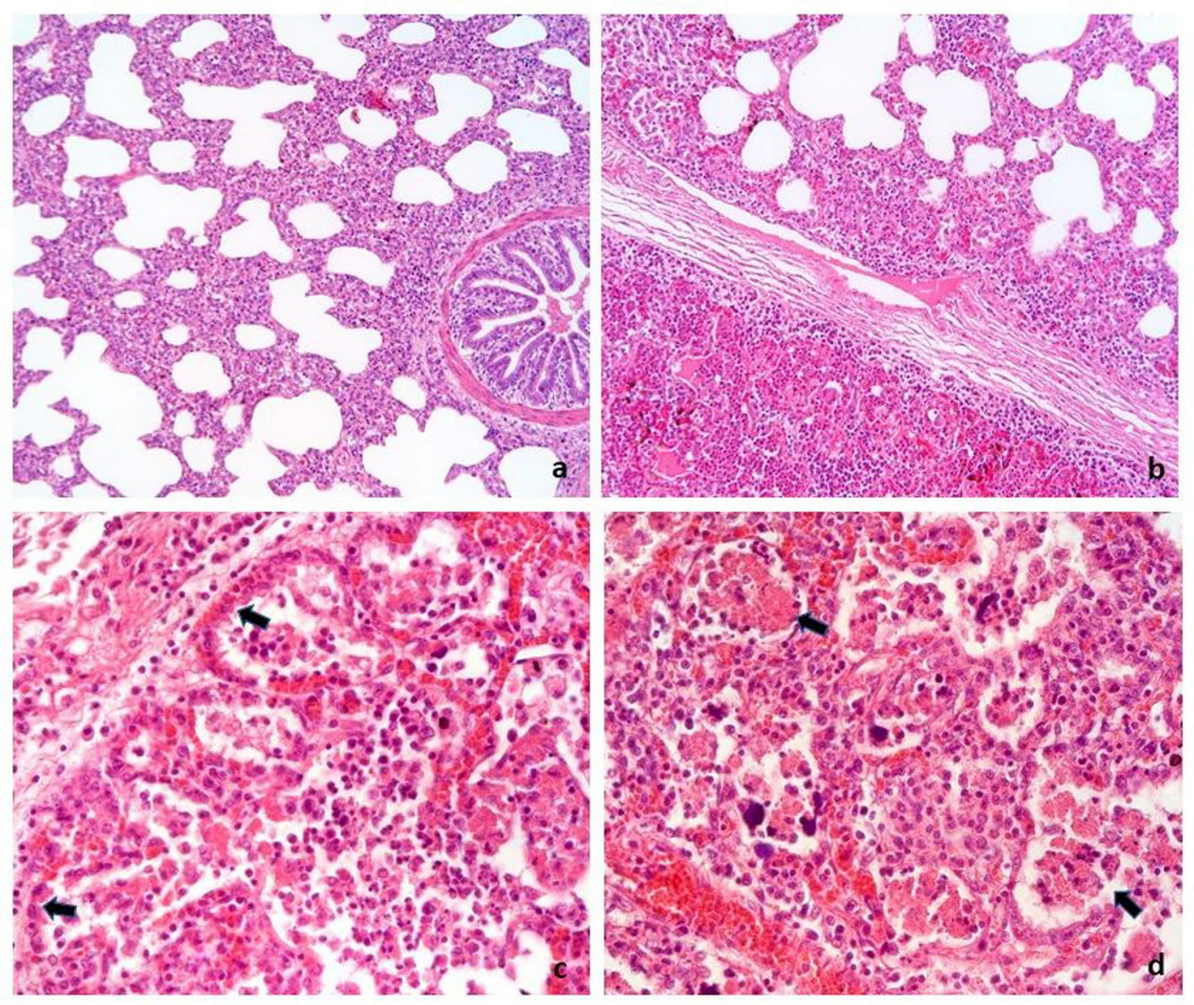
Swine, lung. Histologic features of the main pneumonia patterns recognized in 79 cases of PRDC. **(A)** Interstitial pneumonia characterized by irregular thickening of alveolar septa by inflammatory infiltrate. **(B)** Interstitial pneumonia (top of the figure) associated with bronchoalveolar exudate (bottom of the figure) of neutrophils (bronchopneumonia). **(C,D)** Proliferative and necrotizing pneumonia characterized by hyperplasia of type II pneumocytes (arrow in **C**) and necrosis of macrophages exudate in alveoli (arrow in **D**). H&E. **A,B**: 16×; **C,D**: 40 × .

**Figure 3:**
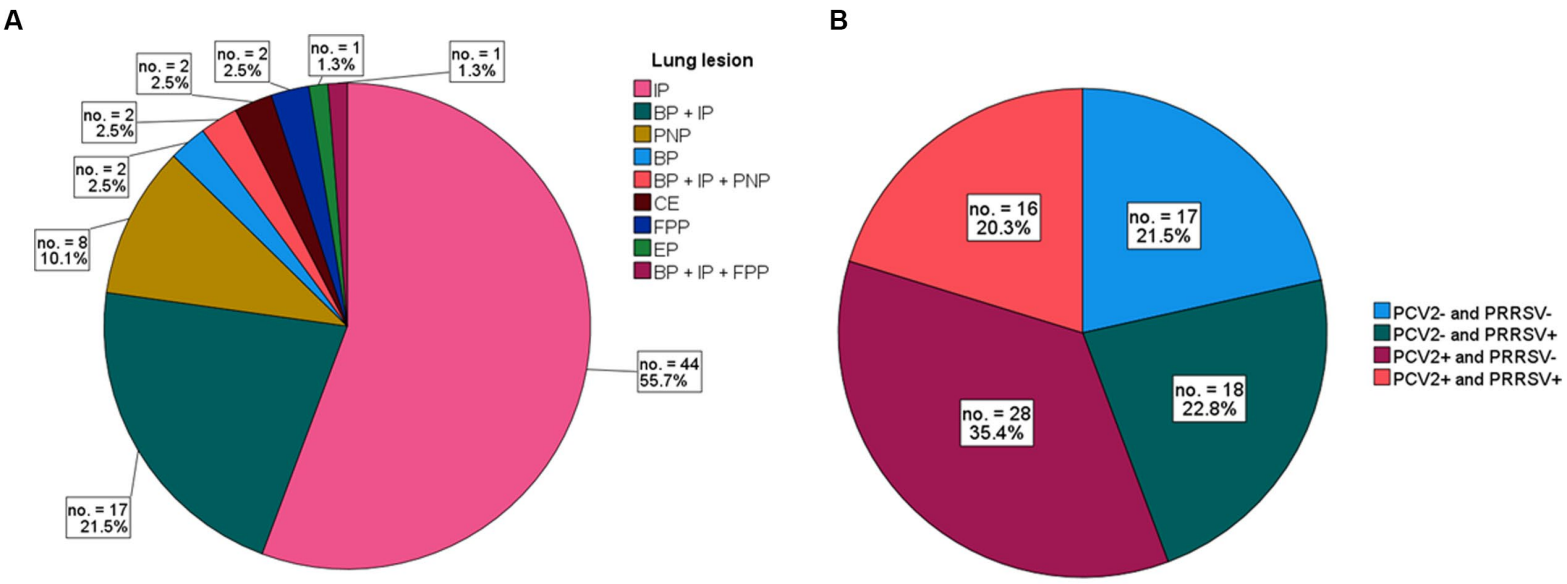
**(A)** Frequency of lung lesions in 79 cases of PRCD. Cases are grouped by single type of pneumonia or their associations. Interstitial pneumonia (IP) alone or associated with other pneumonias accounts for 82% of cases. **(B)** Frequency of PCV2 and/or PRRSV-positive or -negative stain in 79 cases of PRDC.

The following histologic associations were recorded: 44 cases (55.7%) exhibited pure interstitial pneumonia (illustrated in Figure 2A); 17 cases (21.5%) had bronchopneumonia associated with interstitial pneumonia (Figure 2B); 8 cases (10.1%) showed evidence of proliferative and necrotizing pneumonia (PNP) (Figures 2C,D); 2 cases (2.5%) had PNP in conjunction with interstitial pneumonia and exudative bronchopneumonia; 2 cases (2.5%) had fibrinous pleuropneumonia; 2 cases (2.5%) exhibited pure bronchopneumonia; 1 case (1.3%) had bronchopneumonia along with interstitial pneumonia and pleuropneumonia; 2 cases (2.5%) displayed only congestion and pulmonary hemorrhages; and 1 case (1.3%) had enzootic pneumonia (Figure 3).

**Figure 4:**
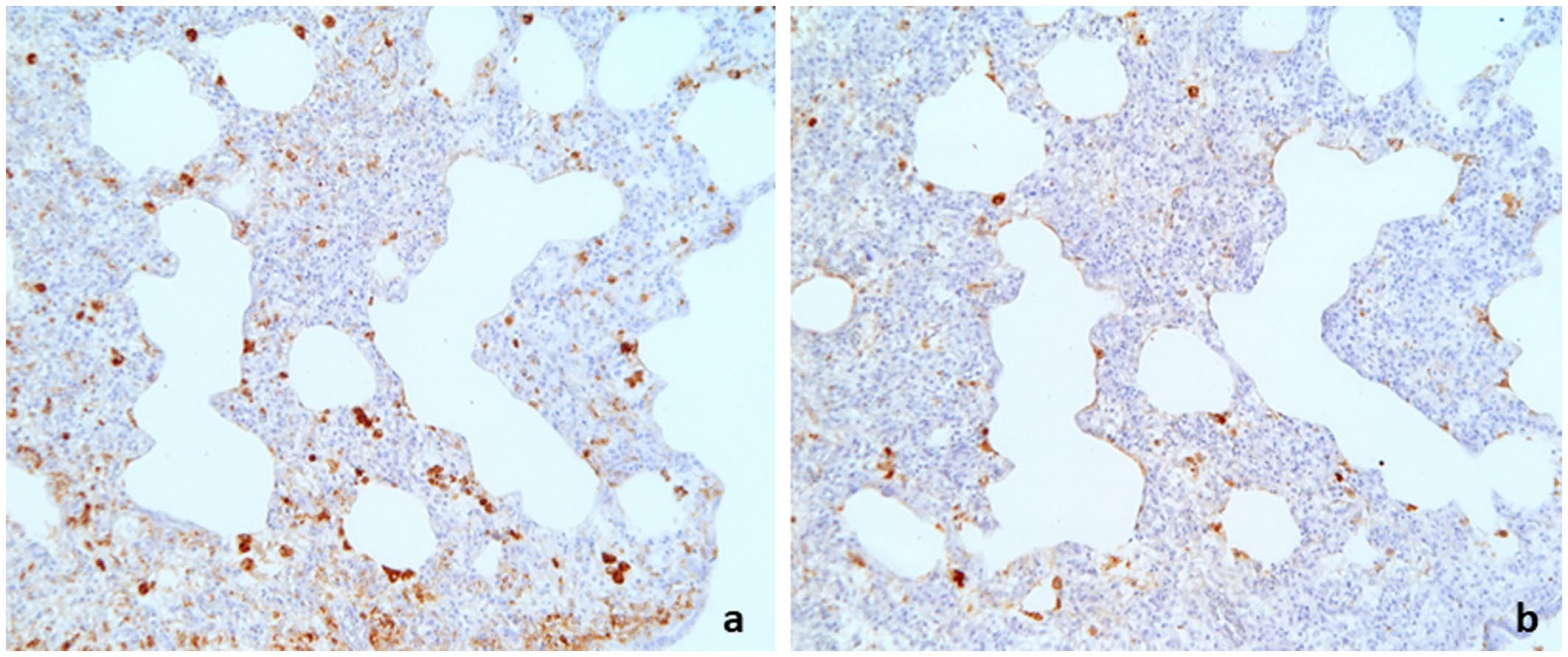
Swine lung. Interstitial pneumonia showing double positive immunostaining to PCV2 **(A)** and PRRSV **(B)**. **(A)**: IHC to PCV2, 16×; **(B)**: IHC to PRRSV, 16×.

The results of the IHC staining on 79 cases of PRDC revealed that 44 (55.7%) tested PCV2 positive in the lung or in at least one out of the available lymphoid tissues, while 34 (43.0%) cases tested positive for PRRSV. In 16 (20.3%) of the total lung tested, both PCV2 and PRRSV were identified (Figure 4). In 17 cases (21.5%), neither virus was detected. Table 1 summarizes the IHC findings for each lung lesion in these cases.

**Table 1:** Summary of results of PCV2 and PRRSV immunohistochemistry (IHC) associated with the pulmonary lesions.

Lung lesion*	No. of cases	Immunohistochemistry			
		Positive			Negative
		PCV2	PRRSV	PCV2 and PRRSV	PCV2/PRRSV
BP	2	0	1	1	0
BP + IP	17	7	5	0	5
BP + IP + PNP	2	0	2	0	0
BP + IP + FPP	1	1	0	0	0
IP	44	17	6	11	10
CE	2	1	0	0	1
FPP	2	0	0	1	1
PNP	8	2	3	3	0
EP	1	0	1	0	0
total	79	28	18	16	17

*BP, bronchopneumonia; IP, interstitial pneumonia; PNP, proliferative and necrotizing pneumonia; CE, congestion and hemorrhage; FPP, fibrinous pleuropneumonia; EP, enzootic pneumonia.

Among the 79 cases, 10 (12.7%) showed lung lesions associated with PNP (alone or in conjunction with interstitial pneumonia and exudative bronchopneumonia), of which 5 (50.0%) were PCV2-negative and PRRSV-positive, 2 (20.0%) were PCV2-positive and PRRSV-negative, and 3 (30.0%) were double positive for both.

**Figure 5:**
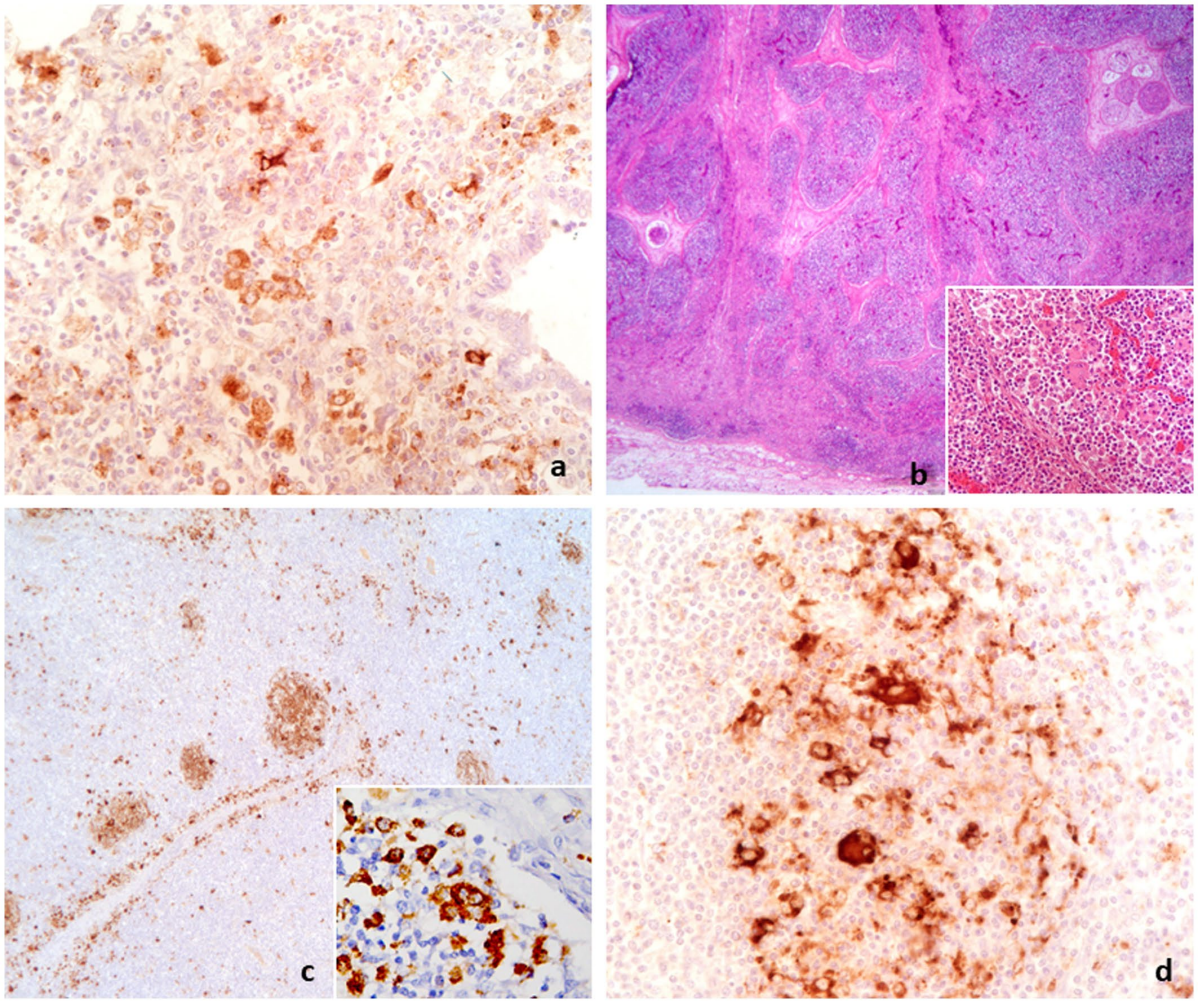
Diagnostic path conclusive for PCV2-SD. **(A)** PCV2-positive macrophages in lung with interstitial pneumonia associated with lymphoid depletion in lymphoid tissue as in lymph node **(B)**. Inset in **(B)**: collection of epithelioid and giant cells in lymph node. **(C)** Depleted lymph node showing PCV2 antigen in residual follicles **(C,D)** and in epithelioid and giant cells (inset in **C**). **(A,D)**: IHC to PCV2, 40×; **(B)**: H&E, 2.5×, inset 40×; **(C)**: IHC to PCV2, 6.3×, inset 40×.

**Figure 6:**
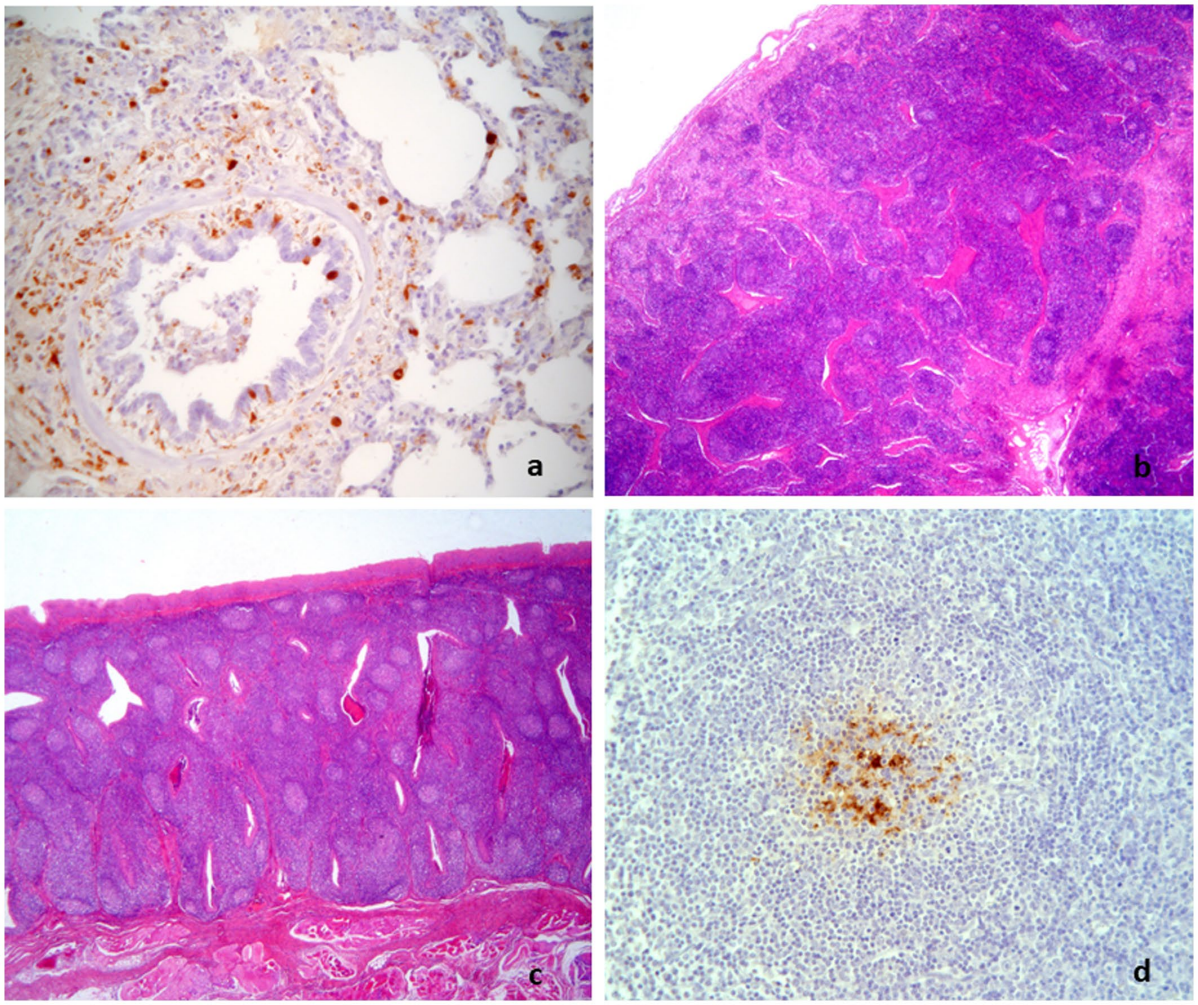
Diagnostic path conclusive for PRDC/PCV2-positive. Interstitial pneumonia with PCV2-positive macrophages **(A)** associated with hyperplastic lymphoid tissues [lymph node in **(B)** and tonsil in **(C)** showing PCV2-positive histiocytes in few normal and polarized follicles **(D)**]. **(A,D)**: IHC to PCV2, 25×; **(B,C)**: H&E, 2.5×.

Among the 44 subjects with lymphoid tissues that tested PCV2-positive through IHC, 28 cases (63.6%) exhibited lesions compatible with lymphocytic depletion and showed IHC positivity for PCV2 in at least one of the lymphoid tissues examined. These cases were classified as PCV2-SD (Figure 5). In the remaining 16 out of 44 cases, in addition to PCV2-positive lung lesions, no lymphoid tissue lesions or only mild to moderate lymphoid hyperplasia were detected. In these cases, slight positivity to the PCV2 antigen was detected within the cytoplasm of centrofollicular dendritic cells in at least one of the lymphoid tissues examined. These cases were classified as PRDC/PCV2-positive (36.4%) (Figure 6).

**Figure 7:**
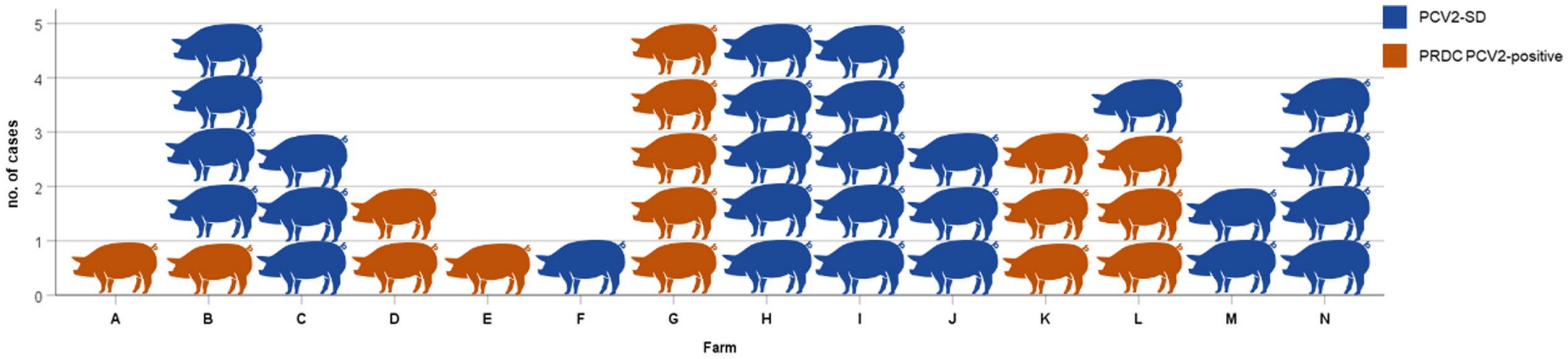
PCV2-SD or PRDC/PCV2-positive cases in each 14 PCV2-positive (A–N) farms. Considering the final diagnosis on the flock, in herds C, F, H, I, J, M, and N it is concluded PCV2-SD. A diagnosis of PCV2-SD is concluded also in farms B and L because at least one pig in the group displayed such final diagnosis. A final diagnosis of PCV2-SD was not reached in herds A, D, E, G, and K, where only PRDC/PCV2-positive was identified.

The results of the classification and definition of individual cases in PCV2-SD and PRDC/PCV2-positive categories were subjected to further analysis by grouping them according to the farm of origin, as shown in Figure 7. A diagnosis of PCV2-SD was established in those farms where all subjects displayed systemic involvement (farms C, F, H, I, J, M, and N in Figure 7). Additionally, a diagnosis of PCVD-SD was made in farms B and L (Figure 7) when at least one pig in the group displayed such final diagnosis. However, a final diagnosis of PCVD-SD could not be reached in herds A, D, E, G, K, where only PRDC/PCV2-positive cases were identified.

**Figure 8:**
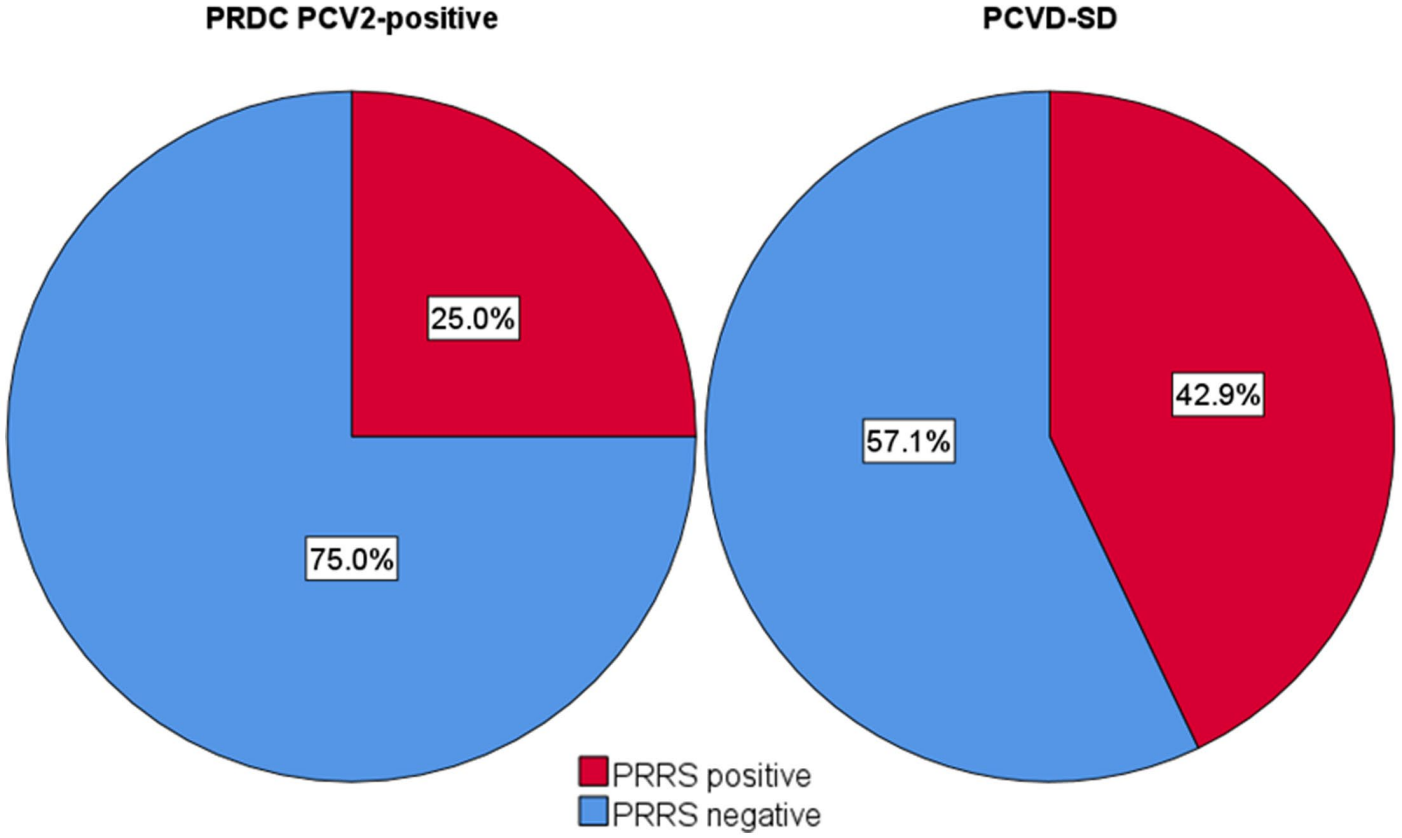
Frequency of PRRSV positive and negative pneumonias in PCV2-SD and PRDC/PCV2-positive cases.

In the 44 PCV2-positive pigs, 42.9% of the PCV2-SD cases (12/28) showed IHC positivity for PRRSV in the lung tissue, while 25.0% of PRDC/PCV2-positive cases (4/16) showed double positivity for PCV2 and PRRSV (Figure 8).

## 4. Discussion

Porcine Respiratory Disease Complex (PRDC) is a high-morbidity clinical condition affecting pigs over 4 weeks of age, exhibiting respiratory signs often unresponsive to antibiotics. This disease is frequently associated with a wide spectrum of lung lesions that are caused by the synergistic effect of both bacterial and viral microorganisms. In this study, interstitial pneumonia and mixed forms of both exudative bronchopneumonia and interstitial pneumonia were the most frequently (77.2%) observed microscopic lesions.

Viral infections, such as Porcine Circovirus type 2 (PCV2) and Porcine Reproductive and Respiratory Syndrome Virus (PRRSV), reaching the lung through the hematogenous route, are frequently associated with interstitial pneumonia in pigs. At the same time neutrophilic and macrophagic exudation in bronchiolo-alveolar spaces provides an indication of secondary bacterial infection further complicating the lung lesions (12). Microscopic pulmonary lesions, identified by the criteria used to diagnose PRDC, suggest that viral infections are the primary cause, exacerbated by secondary bacterial infections (1, 39).

In our study, the results of the immunohistochemistry (IHC) assay for PCV2 and PRRSV confirmed the link between the microscopic appearance of the lesions and their viral origin, as the antigens of the two viruses were identified in 78.5% of the overall cases and in 49 out of 64 cases (77%) of interstitial pneumonia included as single or combined diagnosis (Table 1).

PCV2 and PRRSV, although not strictly considered respiratory pathogens, are well-known viruses heavily involved in swine respiratory disease clinical manifestation (1). In 20.3% of the cases (16 out of 79) presented here, co-infection between PCV2 and PRRSV was identified through double-positive PCV2 and PRRSV results in lungs, as determined by IHC. PRRSV infection is known to act as a co-factor that exacerbates lesions and generates the clinical signs of PCVD (30, 40), and co-infection between PCV2 and PRRSV is a well-established phenomenon (27–31).

Consistent with previous research (28, 41), the majority of proliferative and necrotizing pneumonia (PNP) cases in this study were linked to PRRSV infection and the presence of viral antigen within lung lesions. Moreover, a significant proportion of PNP cases (3/10, 30%) are double-positive for PCV2 and PRRSV in the lung. PNP is a form of interstitial pneumonia characterized by hypertrophy and hyperplasia of type II pneumocytes associated with the presence of necrotic debris within alveolar spaces (42). PRRSV and PCV2 are the main viral agents involved in PNP, considering Aujezsky’s disease virus (ADV) and swine influenza A virus (IAV-S) playing a possible role in co-infections (43). Our study confirms previous findings by Morandi et al. (41), indicating that the severe form of interstitial pneumonia in Italy is primarily associated with PRRSV infection. Additionally, we observed here that co-infection with PCV2 resulted in more severe microscopical lesion.

The sampling method used in this study, which included a variety of lymphoid tissues in addition to the lung, allowed us to diagnose PCV2-SD in lung PCV2-positive cases by combining the results of lymphoid tissue lesions and IHC positivity. As a result, in 28 out of 44 PCV2-SD cases (63.6%), PCV2-positive stain was found in at least one lymphoid-depleted tissue, including the tonsil, tracheobronchial lymph node, superficial inguinal lymph node, spleen, and ileum, in addition to pulmonary lesions.

The diagnosis of systemic Porcine Circovirus type 2 disease has been well-established since the earliest descriptions of PCV2-related disease (18, 44) and continues to be used today (23). These criteria encompass the triad of clinical signs, lesions, and viral detection in the presence of recognizable lesions. The case series reviewed in this study showed that a higher proportion of cases had evidence of systemic infection despite presenting respiratory signs without severe emaciation and anemia, which are typical of PCV2-associated systemic disease (PCV2-SD). Thus, these cases were classified as PCV2-SD. This finding aligns with the study conducted by Ticó et al. (24), where on the 226 PCV2-positive animals suffering from PRDC, 184 cases had PCV2 systemic infection, suggesting that PCV2-LD is a negligible condition and possibly part of a systemic manifestation of infection.

The introduction of PCV2 vaccines has generated significant modifications in the epidemiology of the disease (45). As a result, the severity of disease presentation has decreased over time, while the prevalence of vague clinical signs has increased in affected animals (46). Nevertheless, although PCV2 vaccines are among the most effective used in swine herds, cases of PCV2-SD have been described in vaccinated farms (47, 48). The primary contributing factor to this phenomenon can be attributed to inappropriate vaccination protocols, whereby the timing of vaccine administration is either delayed or premature. Notably this is not driven by the circulation of new viral strains capable of evading vaccine-induced protection (49), but rather the disruption of maternal-derived immunity (23).

Additionally, it is noteworthy that PCVD may occur after a PCV2 infection triggered by co-infections with other viruses and bacteria (50). In this context, the Porcine Reproductive and Respiratory Syndrome Virus (PRRSV) has been identified as a key pathogen in co-infections in pigs (51, 52). In the current study, 7 out of the 22 farms had clinical PRRSV recrudescence reported in anamnesis, and 12 out of 28 pigs with a final diagnosis of PCV2-SD were also found to be PRRSV-positive in the lungs. Furthermore, experimental studies have demonstrated that co-infections with PCV2 and PRRSV can result in more severe clinical signs and lesions than infections with a single virus (53, 54). This synergism between the two viruses in regulating host innate and adaptive immunity (55–57) can lead to higher replication and pathogenicity of the viruses involved (29, 35, 58, 59). In the field, this also occurs in the case of vaccination against PRRSV in herds infected with PCV2; in fact, vaccination with live attenuated PRRSV vaccine has been shown to enhance PCV2 replication and increase viraemia with a mechanism identical to a PCV2/PRRSV coinfection (60). For the control of clinical forms, it is, therefore, pivotal to know the health status of the herd in order to choose the most suitable vaccination strategy (61).

The diagnosis of PCVD is a herd diagnosis and should be performed in a group of 3–5 animals (50). In our study among positive PCV2 farms (14/22), in 7 sites, all the examined pigs presented the criteria for categorization in PCV2-SD (Figure 7). In 5 herds, all forms observed were referable to PRDC/PCV2-positive, although the data may not be sufficient for a reliable herd diagnosis. Only one subject was conferred from two farms and only two from one, despite the 3-5 needed. On the other hand, in two herds, the results differed among the individual cases examined. However, the evidence of a systemic form of the disease in at least one animal tested supports the diagnostic conclusion of PCV2-SD in the group.

In this study, the individual cases classified PRDC/PCV2-positive (with specific lung lesions and immunohistochemical positive stain in the lung and in at least one of the examined lymphoid districts) were those in which the lymphocyte depletion in lymphoid tissues were not observed (16/44, 36.4%). The occurrence of microscopic lung lesions caused solely by PCV2 infection is rare in experimental conditions, as evidence by the findings of Hoogland et al. (62) and Opriessing et al. (63). However, in field conditions, the situation is considerably more complex, with numerous co-factors, infectious and non-infectious, potentially exacerbating lung damage. Notably, Grau-Roma et al. (64) reported a positive correlation between the PCV2 viral load in serum and tissues, implying a significant role of PCV2 in the onset of Porcine Respiratory Disease Complex (PRDC), even in subclinical cases and in the absence of lymphoid depletion.

Empirical evidence suggests that despite the absence of a definitive diagnosis of systemic Porcine Circovirus type 2 (PCV2) disease, the virus is prevalent in tissues and continues to circulate, invariably compromising the herd’s health.

Our case series reveals a strong association between PCV2 and a considerable percentage of respiratory signs, even when the systemic disease is absent. In such scenarios, where the likelihood of a bacterial origin for Porcine Respiratory Disease Complex (PRDC) is ruled out, detecting PCV2 infection should clearly indicate the need to strengthen control and preventive measures on the farm.

## 5. Conclusion

The results of this study highlight the significance of PCV2, particularly when its antigen is detected in the lung tissue in conjunction with microscopic interstitial pneumonia. The systemic form of PCVD (PCV2-SD) is diagnosed when the PCV2 antigen is present, given its strong association with both pulmonary issues but also lymphoid depletion. To avoid underestimating the potential harm caused by PCV2, it is imperative to employ appropriate sampling techniques to diagnose the systemic form of the disease, even in the absence of other clinical signs. Despite the outdated notion of PCV2-LD (23), PCV2, along with PRRSV, remains a prevalent infection linked to PRDC and has a negative impact on herd health. Co-infections can exacerbate the pathological condition, necessitating control measures based on pathological and analytical evidence.

## Data availability statement

The raw data supporting the conclusions of this article will be made available by the authors, without undue reservation.

## Ethics statement

No ethics approval is required. The study was conducted in accordance with the local legislation and institutional requirements.

## Author contributions

GD’A and GS: conceptualization and writing—original draft preparation. GD’A, GS, AL, and MO: methodology. FO and LVM: validation. GD’A, GS, and LVM: investigation. GD’A, GS, and FO: data curation. MO and FO: writing—review and editing. NT, AL, and LuM: visualization. GS: supervision. NJ and GL: project administration. All authors contributed to the article and approved the submitted version.

## Conflict of interest

NJ, NT, and GL were employed by Boehringer Ingelheim Animal Health Italia SpA.

The remaining authors declare that the research was conducted in the absence of any commercial or financial relationships that could be construed as a potential conflict of interest.

## Publisher’s note

All claims expressed in this article are solely those of the authors and do not necessarily represent those of their affiliated organizations, or those of the publisher, the editors and the reviewers. Any product that may be evaluated in this article, or claim that may be made by its manufacturer, is not guaranteed or endorsed by the publisher.
